# A Study on the Pattern of Ocular Injuries and Their Visual Outcomes Following Road Traffic Accidents

**DOI:** 10.7759/cureus.83985

**Published:** 2025-05-12

**Authors:** Jennifer Gagrai, Rahul Prasad, Varsha Bhagat, Sindhu Kumari, Nishtha Mishra

**Affiliations:** 1 Department of Ophthalmology, Regional Institute of Ophthalmology, Rajendra Institute of Medical Sciences, Ranchi, IND

**Keywords:** ocular injuries, ocular trauma, road traffic accidents, unilateral blindness, vision acuity, visual outcomes

## Abstract

Background: Ocular trauma is a major contributor to preventable monocular visual morbidity, which includes blindness and visual impairment. It is the most prevalent cause of unilateral blindness, accounting for unilateral visual loss and blindness worldwide. Thus, the study evaluated ocular injuries resulting from road traffic accidents (RTAs), along with clinical findings and the visual outcomes in affected patients.

Materials and methods: It was a prospective observational study. The study was conducted at the Department of Trauma & Emergency of the Regional Institute of Ophthalmology, Rajendra Institute of Medical Sciences (RIMS), Ranchi, Jharkhand, India. Overall, 292 patients were enrolled in the study.

Results: The average age of all the included participants was 42 ± 10.5 years. A total of 234 (80.2%) of participants were male. It was discovered that closed-globe injuries had superior visual acuity than open-globe injuries. The majority of affected eyes achieved a best corrected visual acuity of 6/6. Visual acuity between both eyes was found to be highly statistically significant at a p-value less than 0.001.

Conclusion: The study concluded that men experience more trauma in RTAs. Statistically significant results have been observed among participants in terms of visual acuity between the right and left eyes. Various fractures involving the orbital walls were noted.

## Introduction

One of the top 10 causes of death worldwide is road accidents. India accounts for 11% of all accident-related deaths worldwide, according to a recent World Health Organization (WHO) report, and the rate will rise over time as the number of vehicles increases [1]. Ocular trauma is a substantial contributor to preventable monocular visual morbidity, encompassing blindness and visual impairment. Road traffic accidents (RTAs) are a major contributor to visual impairment and ocular damage, especially in developing nations such as India. In these areas, RTAs are responsible for almost 85% of all fatalities and 90% of disability-adjusted life years (DALYs) lost as a result of traffic-related injuries [2].

Factors such as rapid urbanization, increased motorization, inadequate road engineering, low awareness levels, lack of injury prevention programs, and poor enforcement of traffic laws contribute to the high incidence of RTAs. In developing nations, RTAs cause over 85% of all fatalities and 90% of DALYs lost. RTA-related injuries and deaths are a serious public health concern [2]. RTA-related eye injuries, which frequently cause some degree of sight loss, come at a high cost to society and the victims [3].

Damaged areas of the eye include the orbital wall, conjunctiva, cornea, sclera, eyelids, lacrimal canaliculi, and extraocular muscles. Potential injuries include uveal prolapse, traumatic cataracts, vitreous hemorrhage, choroidal rupture, vitreous loss, retinal detachment, optic nerve avulsion, and globe rupture [3].

Approximately 75% of ocular crises are caused by ocular trauma, which is a prevalent and avoidable cause of vision impairment [4]. One significant risk factor for these injuries is RTAs. One of the most frequent avoidable causes of ocular morbidity and monocular loss of vision is ocular trauma from repetitive trauma injuries [5]. Ocular trauma is the most prevalent cause of unilateral blindness, accounting for over 1.6 million blind individuals worldwide and an additional 19 million having unilateral visual loss [6].

The mandated use of safety seat belts, laminated glass windscreens, child car seats, public education about road safety, wearing helmets, donning seat belts, and donning unbreakable plastic spectacles are some of the preventive strategies to lessen ocular injuries from rear-end collisions. Additionally, crucial to reducing RTAs are clearly visible road signs and markers, particularly those that are illuminated at night [3]. A similar study conducted in Assam to compare several related risk factors and evaluate the visual outcome in patients with ocular injuries who are admitted to a tertiary care hospital after RTA reported that early treatment of eye injuries and the use of protective gear lead to better visual outcomes. The younger age group was most affected, with a male preponderance, alcohol, and two-wheelers being significant risk factors [7].

Proper adherence to protective safety measures and traffic rules can effectively decrease the incidence of ocular trauma. Thus, the study evaluated the ocular injury resulting from RTAs along with clinical findings and the visual outcomes in affected patients.

## Materials and methods

Study design

This was a prospective observational study. The study was conducted at the Department of Trauma & Emergency of the Regional Institute of Ophthalmology, Rajendra Institute of Medical Sciences (RIMS), Ranchi, Jharkhand, India. The study has been conducted for one and a half years, i.e., from October 2022 to April 2024.

Study population

A total of 292 patients were enrolled in the study. The consecutive sampling technique was preferred. The inclusion criteria for patients were all cases of ocular trauma due to RTA presenting for the first time within two weeks in the emergency and outpatient department of the Regional Institute of Ophthalmology, RIMS, Ranchi, who gave consent to participate in the study. The exclusion criteria for patients were old cases of ocular trauma due to RTA treated elsewhere and unstable patients who had sustained an eye injury alongside other serious, life-threatening injuries.

Data collection

Written informed consent was taken from all participants prior to the study after an explanation regarding the gains, risks, and goals of the study in the patient’s own language. All participants were subjected to detailed clinical evaluation before and after the procedure. Patient demographics and history were recorded.

Study procedure

A detailed external ocular examination was done. Investigations like dilated fundus examination, tonometry, B-scan ultrasonography, optical coherence tomography, MRI/CT scan of the brain and orbit, X-ray, and other laboratory investigations were performed. Further, a follow-up has been done for six months. First follow-up was conducted after one month, second follow-up was conducted after three months, and last follow-up was conducted after six months.

Statistical analysis

All the observed findings were noted on a Microsoft Excel sheet (Microsoft Corporation, Redmond, WA), and data were further analyzed using SPSS version 26 (IBM Corp., Armonk, NY). Microsoft Word and Excel were used to generate graphs and tables. Descriptive and inferential statistical analyses were carried out in the present study. Results on continuous measurements were presented as mean ± standard deviation (SD), and results on categorical measurements were presented as a percentage (%). An independent t-test or chi-square test was used to obtain the p-value. P-value was considered significant at less than 0.05.

Ethical clearance

The study was initially compiled with the Declaration of Helsinki, and informed consent was obtained from all the participants. Ethical approval has been granted by the Institutional Ethics Committee of Rajendra Institute of Medical Sciences, Ranchi, India (Memo No.: 68 IEC, RIMS).

## Results

Overall, 292 patients were assessed. Table 1 depicts patient characteristics such as age, gender, involved eye, orbit fracture, and the type of injury of enrolled participants. The average age of all the included participants was 42 ± 10.5 years. A total of 234 (80.2%) of participants were male. Of the patients, 103 (35.2%) had open injury, while 189 (64.7%) patients had closed injury, which means an injury to the eye that is blunt yet leaves the cornea and sclera intact.

**Table 1:** Patients characteristics. Data were presented as either mean ± SD or n (%).

Characteristics	Value
Age (in years)	42 ± 10.5
Male participants	234 (80.2%)
Female participants	58 (19.8%)
Eye involved	
Right eye	142 (48.6%)
Left eye	135 (46.1%)
Both eye	15 (5.1%)
Orbit fracture	35 (11.9%)
Open injury	103 (35.2%)
Closed injury	189 (64.7%)

Out of 292 patients, 209 (71.58%) had lid edema and ecchymosis, 173 (59.25%) has conjunctival foreign body, 124 (42.29%) had chemosis, 141 (48.29%) had suprachoroidal hemorrhage, 38 (13.01%) had corneoscleral tear, 16 (5.48%) had corneal foreign body, 91 (31.16%) had corneal tear, 36 (12.33%) had corneal abrasion, four (1.37%) had foreign body in the anterior chamber, and 107 (36.64%) had hyphaemia. Findings from the anterior segment are shown in Table 2.

**Table 2:** Findings of anterior segment. Data were presented as n (%).

Anterior segment	Value
Lid edema & ecchymosis	209 (71.58%)
Conjunctival foreign body	173 (59.25%)
Chemosis	124 (42.47%)
Suprachoroidal hemorrhage	141 (48.29%)
Corneoscleral tear	38 (13.01%)
Corneal foreign body	16 (5.48%)
Corneal tear	91 (31.16%)
Corneal abrasion	36 (12.33%)
Foreign body in the anterior chamber	04 (1.37%)
Hyphaemia	107 (36.64%)

Out of 292 patients, vitreous prolapse was seen in six (2.05%) patients, vitreous hemorrhage in nine (3.08%), vitreous was clear in 166 (56.85%), and vitreous was not assessed in 111 (38.01%) patients. Macular findings included Berlin's edema in nine (3.08%), macular hole in two (0.68%), and the macula was not assessed in 93 (31.85%) patients. Retinal hemorrhage and retinal detachment were noted in four (1.37%) patients, respectively. In 93 (31.85%) cases, the retina cannot be assessed due to vitreous haze. Also, traumatic neuropathy was observed in 11 (3.77%) patients. Table 3 represents posterior segment findings.

**Table 3:** Findings of the posterior segment. Data were presented as n (%).

Posterior segment		Value
Vitreous	Vitreous prolapse	06 (2.05%)
	Vitreous hemorrhage	09 (3.08%)
	Clear	166 (56.85%)
	Not assessed	111 (38.01%)
Macula	Berlin’s edema	09 (3.08%)
	Macular hole	02 (0.68%)
	Foveal reflex present	188 (64.38%)
	Not assessed	93 (31.85%)
Retina	Normal	191 (65.41%)
	Retinal hemorrhage	04 (1.37%)
	Retinal detachment	04 (1.37%)
	Not assessed	93 (31.85%)
Optic nerve	Normal	188 (64.38%)
	Traumatic optic neuropathy	11 (3.77%)
	Not assessed	93 (31.85%)

Visual acuity was found to perform better in closed globe injuries than in open globe injuries. The distribution of visual acuity at the time of presentation is displayed in Table 4. A total of 82 patients (28.08%) had visual acuity of greater than 6/60, 131 patients (44.86%) varied from 2/60 to 6/60, 55 patients (18.84%) from perception of light (PL)+ to 1/60, and 24 patients (8.22%) did not perceive light in their right eye. A total of 89 patients (30.48%) had visual acuity of at least 6/60, 140 patients (47.95%) varied from 2/60 to 6/60, 46 patients (15.75%) ranged from PL+ to 1/60, and 17 patients (5.82%) could not see light in their left eye. Visual acuity between both eyes was found to be highly statistically significant at a p-value less than 0.001.

**Table 4:** Visual acuity of participants initially. Data were presented as n (%). The chi-square test was used to obtain the p-value. P-value was considered significant at less than 0.05. PL: perception of light.

Visual acuity	No. of participants		Chi-square statistic	p-value
	Right eye	Left eye	21.64	<0.001
PL-	24 (8.22%)	17 (5.82%)		
PL+ to 1/60	55 (18.8%)	16 (15.75%)		
2/60 to 6/60	131 (44.8%)	140 (47.9%)		
>6/60	82 (28%)	89 (30.4%)		

Table 5 depicts best corrected visual acuity (BCVA) at one month, three months, and six months, respectively. The majority of affected eyes achieved a BCVA of 6/6.

**Table 5:** Visual acuity in eyes after one, three, and six months. Data were presented as n (%). An independent t-test was used to obtain the p-value. P-value was considered significant at less than 0.05. HM: hand motion; PL: perception of light; NPL: no light perception.

Visual acuity in the eyes	1^st^ month follow-up			3^rd^ month follow-up			6^th^ month follow-up		
	Right eye	Left eye	p-value	Right eye	Left eye	p-value	Right eye	Left eye	p-value
6/6	153 (52.4%)	157 (53.77%)	0.37	203 (69.52%)	211 (72.2%)	0.23	205 (70.2%)	210 (71.9%)	0.32
6/9	42 (14.3%)	42 (14.3%)	0.5	03 (1.03%)	06 (2.05%)	0.15	10 (3.4%)	08 (2.7%)	0.31
6/12	11 (3.7%)	20 (6.8%)	0.04	05 (1.71%)	05 (1.7%)	0.5	03 (1.03%)	08 (2.7%)	0.06
6/18	01 (0.3%)	02 (0.68%)	0.28	04 (1.37%)	02 (0.68%)	0.2	08 (2.7%)	10 (3.4%)	0.31
6/24	03 (1.0%)	00	-	07 (2.4%)	09 (3.08%)	0.30	07 (2.4%)	04 (1.3%)	0.18
6/36	02 (0.68%)	00	-	05 (1.7%)	07 (2.4%)	0.27	07 (2.4%)	10 (3.4%)	0.23
6/60	01 (0.34%)	04 (1.37%)	0.08	20 (6.8%)	15 (5.14%)	0.19	16 (5.4%)	13 (4.4%)	0.28
5/60	03 (1.03%)	02 (0.68%)	0.32	08 (2.7%)	05 (1.71%)	0.20	04 (1.3%)	05 (1.7%)	0.36
4/60	00	03 (1.03%)	-	03 (1.03%)	06 (2.05%)	0.15	03 (1.03%)	01 (0.34%)	0.15
3/60	01 (0.34%)	04 (1.37%)	0.08	03 (1.03%)	02 (0.68%)	0.32	02 (0.68%)	00	-
2/60	11 (3.7%)	07 (2.40%)	0.16	02 (0.68%)	02 (0.68%)	0.5	00	01 (0.34%)	-
1/60	11 (3.7%)	14 (4.79%)	0.26	02 (0.68%)	01 (0.34%)	0.28	01 (0.34%)	02 (0.68%)	0.28
HM	32 (10.9%)	19 (6.51%)	0.02	06 (2.05%)	02 (0.68%)	0.07	04 (1.37%)	00	-
PL+	06 (2.05%)	03 (1.03%)	0.15	03 (1.03%)	03 (1.03%)	0.5	03 (1.03%)	02 (0.68%)	0.32
NPL	15 (5.14%)	15 (5.1%)	0.5	18 (6.16%)	16 (5.4%)	0.36	19 (6.51%)	18 (6.1%)	0.43

Those who were unable to achieve BCVA were mainly due to post-traumatic ocular complications such as corneal scarring in 63 (21.6%) patients, traumatic optic neuropathy in 11 (3.8%) patients, choroid rupture in 35 (11.9%) patients, cataract in 25 (8.56%) patients, post-traumatic glaucoma in 18 (6.2%) patients, retinal detachment in four (1.4%) patients, and non-closing full-thickness macular hole in two (0.6%) patients. Table 6 shows late complications in participants.

**Table 6:** Complications in participants. Data were presented as n (%).

Late complications	Number of participants (n = 292)
Corneal scarring	63 (21.6%)
Traumatic optic neuropathy	11 (3.8%)
Choroid rupture	35 (11.9%)
Cataract	25 (8.56%)
Post-traumatic glaucoma	18 (6.2%)
Retinal detachment	04 (1.4%)
Non-closing full-thickness macular hole	02 (0.6%)

## Discussion

In the present study, a total of 292 patients were enrolled. The purpose of the investigation was to comprehend the clinical signs, contributing variables, and possible treatments to lessen eye injuries from RTAs.

The age distribution of the subjects was 42 ± 10.5. In our survey, there were 80.2% men and 19.8% women; the male-to-female ratio was 4.03:1. This outcome almost matches the research findings by Eagling et al., who found the male-to-female ratio to be 6.5:1 [8]. According to related research conducted in 1987 by Jain et al., the incidence was 30.7% for females and 69.3% for men [9]. Young men are more likely to get eye injuries [10]. The young age group was shown to have the highest prevalence of eye damage. This is consistent with the research conducted by Dandona et al. [11].

The male preponderance in RTAs has always been noteworthy; this might be explained by the high mobility and activity of men and the higher occurrence of male drivers in India compared to female drivers. Additionally, men are more likely than women to engage in aggressive outdoor activities. Because of this, men are more likely to suffer eye injuries.

A total of 142 (48.63%) patients were involved in injury in the right eye due to RTA, and 135 (46.1%) were involved in injury in the left eye. Injuries in both eyes were seen in 15 (5.1%) patients.

Of the individuals in our research, 35 (11.9%) experienced an orbital fracture. A study by Kamath et al. on ocular fractures in a tertiary healthcare facility obtained similar results [12].

The most frequent findings were retinal tears in four (1.37%) patients and vitreous hemorrhage in nine (3.08%) patients, each followed by vitreous prolapse in six (2.05%) and Berlin's edema in nine (3.08%) patients. In the posterior section, vitreous hemorrhage was shown to be the most prevalent finding in a study by Chugh et al. [13].

Twelve individuals with relative afferent pupillary defect (RAPD) had a significant visual impairment. The pallor of the disc did not appear in those individuals right after the injury. However, they eventually became pale. This study reports that traumatic optic neuropathy affects 3.77% of the population, compared to 0.6% in an epidemiological study conducted in 2007 by Vats et al. on ocular trauma in an urban slum population in Delhi [14]. This indicates that RTA is more common in cases of optic neuropathy than general ocular damage.

The majority of affected eyes achieved a BCVA of 6/6. Those who were unable to achieve this were due to post-traumatic ocular complications such as corneal scarring (40%), cataracts (15.82), post-traumatic glaucoma (11.39%), traumatic optic neuropathy (7%), choroid rupture (22%), non-closing macular hole (1.26%), and retinal detachment (3%).

The study had several limitations, such as being single-centric and having a brief study period. There were some challenges faced during the screening of the participants. Also, it was a bit difficult to collect the data on follow-up periods due to multiple patient visits. The study mainly focused on ocular injuries caused by RTAs. Hence, not focused on trauma or its types. Thus, multivariate analysis was also not performed to find the association of visual outcomes with trauma type or etiology.

## Conclusions

The study concluded that men experience more trauma in RTAs. Statistically significant results have been observed among participants in terms of visual acuity between the right and left eyes. Various fractures involving the orbital walls were noted. The patients who had not achieved BCVA were mainly due to late complications. The patient needs to be evaluated right away and sent to an ophthalmologist for proper care. Thus, routine ophthalmic evaluation in all RTA victims, regardless of severity, is important.
